# Analysis of RNAseq datasets from a comparative infectious disease zebrafish model using GeneTiles bioinformatics

**DOI:** 10.1007/s00251-014-0820-3

**Published:** 2014-12-13

**Authors:** Wouter J. Veneman, Jan de Sonneville, Kees-Jan van der Kolk, Anita Ordas, Zaid Al-Ars, Annemarie H. Meijer, Herman P. Spaink

**Affiliations:** 1 Institute of Biology, Leiden University, Einsteinweg 55, 2333 CC Leiden, The Netherlands; 2 Life Science Methods BV, J.H. Oortweg 19, 2333 CH Leiden, The Netherlands; 3 Computer Engineering, Delft University of Technology, Mekelweg 4, 2628 CD Delft, The Netherlands

**Keywords:** RNA deep sequencing, Host pathogen interaction, Zebrafish, *Staphylococcus epidermidis*, *Mycobacterium marinum*, Differential splicing

## Abstract

We present a RNA deep sequencing (RNAseq) analysis of a comparison of the transcriptome responses to infection of zebrafish larvae with *Staphylococcus epidermidis* and *Mycobacterium marinum* bacteria. We show how our developed GeneTiles software can improve RNAseq analysis approaches by more confidently identifying a large set of markers upon infection with these bacteria*.* For analysis of RNAseq data currently, software programs such as Bowtie2 and Samtools are indispensable. However, these programs that are designed for a LINUX environment require some dedicated programming skills and have no options for visualisation of the resulting mapped sequence reads. Especially with large data sets, this makes the analysis time consuming and difficult for non-expert users. We have applied the GeneTiles software to the analysis of previously published and newly obtained RNAseq datasets of our zebrafish infection model, and we have shown the applicability of this approach also to published RNAseq datasets of other organisms by comparing our data with a published mammalian infection study. In addition, we have implemented the DEXSeq module in the GeneTiles software to identify genes, such as glucagon A, that are differentially spliced under infection conditions. In the analysis of our RNAseq data, this has led to the possibility to improve the size of data sets that could be efficiently compared without using problem-dedicated programs, leading to a quick identification of marker sets. Therefore, this approach will also be highly useful for transcriptome analyses of other organisms for which well-characterised genomes are available.

**Electronic supplementary material:**

The online version of this article (doi:10.1007/s00251-014-0820-3) contains supplementary material, which is available to authorized users.

## Introduction

In our previous research, we have used zebrafish larval infection models to study the transcriptome response to infection by several pathogens (Ordas et al. 2011; Stockhammer et al. 2010; van der Vaart et al. 2013; van Soest et al. 2011; Veneman et al. 2013). In addition, we have tested the response of zebrafish larvae to infection by the opportunistic bacterium *Staphylococcus epidermidis* as a model for biomaterial-associated infections that are often caused by this species in clinical practise (Boelens et al. 2000; Broekhuizen et al. 2008; Busscher et al. 2012; Zaat et al. 2010). These studies have led to a high throughput model that resulted in a large set of RNAseq data sets highlighting a new bottleneck in our research: the fast and user-friendly analysis of large datasets that can be easily visualised for comparative purposes.

In the analysis of our former transcriptome data sets, there was a need for specialised scripting languages to quickly find good marker genes for the disease. We used an existing visualisation program, Integrative Genomics Viewer (IGV) (Robinson et al. 2011), that shows the data solely along a line representing the genome, thereby requiring zooming in to view the aligned reads. IGV and many other open source visualisation programs, such as MapView (Bao et al. 2009), Tablet (Milne et al. 2010), GenoViewer (Laczik et al. 2012) and BamView (Carver et al. 2013), also require the user to scroll or manually search for other genes to bring these into focus; and an overview of a selection of genes based on alignment results is not available. Finally, most of these data visualisation programs do not allow the export of presented visual results, other than taking screenshots. In our previous analysis of RNAseq data, we were reliant on manual counting of reads as guided by the IGV viewer (Veneman et al. 2013).

In this paper, we used the transcriptome data set obtained from the zebrafish high throughput screening system for *S. epidermidis* infection (Veneman et al. 2013) as a case study to optimise and automate the data analysis pipeline. Using the resulting software package, we also went further and added a larger RNAseq data set from *Mycobacterium marinum* infection data for comparisons of specificity of the transcriptome responses. We also integrated the DEXSeq algorithm that can be used to give an estimate of probability of the occurrence of differential splicing. This has led to the identification of genes that are differentially spliced after a microbial infection in zebrafish larvae. Finally, we wanted to include a comparison with whole organism infection data in other vertebrate species. Unfortunately, there are still few available RNAseq data for this that can be mapped on Ensembl genome data; and as a result, we have only been able to compare our zebrafish infection data with the RNAseq data from a bovine digital dermatitis (BDD) model as published by Scholey et al. 2013 (Scholey et al. 2013). However, the results are sufficient to show that our approach makes also such interspecies comparisons of RNAseq datasets very easy and this can quickly lead to conclusions on conserved immune responses, even in comparisons between very different fish and mammalian infection models.

## Material and methods

### Bacterial strains and growth conditions

The *S. epidermidis* strain O-47, containing a pWVW189-derived mCherry expression vector (De Boer L. unpublished), was grown as described in Veneman et al. (2013). The *M. marinum* strain E11 was grown as described in Carvalho et al. 2011 (Carvalho et al. 2011). Two reaction vials with 1 ml of the culture were centrifuged at 14,680 rpm for 1 min. The pellets were combined and washed three times with 1 ml phosphate-buffered saline (PBS). Suspensions were prepared based on the optical density at 600 nm and by plating and colony-forming unit (cfu) determination. The inoculates were suspended in 2 % polyvinylpyrrolidone 40 (PVP_40_, CalBiochem) to 2.0 × 10^7^ or 3.0 × 10^7^ cfu/ml.

### Zebrafish husbandry

Zebrafish were handled in compliance with animal welfare regulations and maintained according to standard protocols (http://ZFIN.org). Embryos were grown at 28 °C in egg water (60 μg/ml Instant Ocean Sea Salt, Sera Marin). The egg water was refreshed every day.

### Experimental outline

Infection experiments were performed with mixed egg clutches from wild-type ABxTL strain zebrafish. Embryos were staged at 2 h post-fertilisation by morphological criteria; and 20 cfu of the mCherry-expressing *S. epidermidis* O-47 or 30 cfu of the mCherry-expressing *M. marinum* E11 bacteria, suspended in 2 % PVP_40_, were injected into the yolk. Automated microinjections were performed as described in (Carvalho et al. 2011). At 5 days post-fertilisation, embryos (N ~100) were collected from the 2 h post-fertilisation injected and non-injected group, snap frozen in liquid nitrogen and stored at −80 °C for RNA isolation.

### RNA deep sequencing

RNA isolation was performed as described in Veneman et al. (2013). A total of 3 μg of RNA was used to make RNAseq libraries using the Illumina TruSeq RNA Sample Preparation Kit v2 (Illumina Inc., San Diego, USA). In the manufacturer’s instructions, two modifications were made. In the adapter ligation, step 1 μl instead of 2.5 μl adaptor was used. In the library size selection step, the library fragments were isolated with a double Ampure XP purification with a 0.7× beads to library ration. The resulting mRNAseq library was sequenced using an Illumina HiSeq2000 instrument according to the manufacturer’s description with a read length of 2 × 50 nucleotides. Image analysis and base calling were done by the Illumina HCS version 1.15.1. The raw RNAseq data have been deposited in the NCBI GEO database with the accession numbers GSE42846, GSE44351 and GSE57792.

### Data analysis

GeneTiles was used for quantification and visualisation of the RNAseq data. When using GeneTiles, the complete data processing pipeline, including the used parameters, is available for download. This enables the user to perform the same analysis locally or try small modifications (for bioinformaticians).

Here, we give a quick list of the used programs and their function within our current pipeline. A detailed explanation including the used parameters is available in Online Resource 2.

In order of use in GeneTiles:

1. Bowtie2 (Langmead and Salzberg 2012) is used to align the reads in the fastq file to the genome (obtained from Ensembl). Bowtie2 generates SAM files that contain the reads together with the location on the genome. Upon multiple hits, the best quality hit is selected or upon a tie of multiple best hits, the reads are randomly distributed (the manual of Bowtie2 is referred to for other default behaviour).
2. Samtools (Li et al. 2009) is used to convert and compress the SAM files into a binary BAM file.
3. Samtools is furthermore used to sort the reads in the BAM files based on the aligned read location in the genome, resulting in a sorted BAM file.
4. The BAM files is indexed to be able to quickly find the aligned reads based on a location in the genome, i.e. to be able to quickly search the BAM file. The index is saved as a BAI file.
5. Using the available annotation from Ensembl, we can search the BAM file for reads within a gene. All reads that at least partially fall within the gene exon and intron regions are counted once. This is done with a python script which consists of the combination of HTseq and pysam (Anders et al. 2014). The output of this script is a tab-separated file (tsv) containing the read counts per gene.
6. We used DESeq, an R-script, to perform statistical analysis. DESeq is used to normalise the reads using a DESeq scaling factor, computed as the median of the ratio, for each gene, of its read count over the geometric mean across samples. Then variance and average of the measurement compared to the control is expressed as a *P* value, by calculating the dispersion per gene using DESeq. The size factors, as well as the *P* values, are stored in ‘tsv’ files.
7. Using scripts, similarly as in step 5, also the input files for DEXSeq can be generated. DEXSeq requires:
  - A ‘gtf’ file containing the experiment design.
  - For all samples, a ‘txt’ file containing the counts obtained for the mapping data in the .SAM files.
  - Genome annotation (from Ensembl) in a ‘gff’ file.

Scripts to obtain these files are also available in the Supplement.

8. Using DEXSeq, another R-script perform a more complex statistical analysis; we can look at the reads within exons and compare the variance and average per exon between measurement and control groups of the samples. DEXSeq uses binning, where exons are cut into bins, based on known exon boundaries. When a read overlaps multiple bins, it is counted in each bin. Per bin, based on the annotation, two comparisons can be made, a comparison between the same exon bins in different samples (groups) and a comparison between an exon bin and its neighbour exon bins within the same group of samples. Note that, therefore, DEXSeq requires at least two groups containing at least two samples. Based on both comparisons, a likelihood test is performed resulting in a *P* value. More details are available in Anders et al. 2012 (Anders et al. 2012). The output of size factors and *P* values are stored as ‘tsv’ files.
9. Using a script, all tsv files are combined into an excel file available for download, e.g. per experiment, chromosome, per filtered results of most significant reads or highest ratio between measurement and control. In addition, an index is built for fast visualisation online (closed source).

Through the website of Wikipathways [http://wikipathways.org/index.php/Download_Pathways], the SVG images were downloaded on the GeneTiles server. Using Javascript, on the client side and within the SVG images, the gene boxes are given a background colour based on a user selection, e.g. *P* value or ratio. For this, the genes and/or proteins are matched to their Ensembl references on the selected genome. In addition, also the human pathways are searched for find homologs of genes using Ensembl biomart. Using these homologs, predictions of homolog pathways can be accessed; this enables to search a larger set of pathways contained in the human section of Wikipathways. It should be noted that using the human pathways to find information about zebrafish biology should be treated with caution and can only lead to suggestions for further investigation.

## Results and discussion

### Software design

RNAseq data, containing tens of millions of reads, is mostly processed using scripts. After processing, a selection of reads is analysed using RNAseq viewers. Directly browsing the processed RNAseq data is difficult due to the large dynamic range of length scales of reads (50 bp), exons (~200 bp), introns (~3 kb), genes (~20 kb) and chromosomes (~65 Mb). Using a minimal size of 1 pixel per read, a computer screen allows only for ~50 kb to be visible. In addition, most RNAseq viewers show introns at the same scale as exons, which in most experiments means that 90 % of the visible sequence data does not display aligned reads. We created an online viewer, GeneTiles (www.Genetiles.com), that does allow for browsing all the aligned reads, while eliminating almost completely the need for user intervention (such as zooming in). The genes in a chromosome are visible as tiles in a 2D array. The tile colour and intensity are a measure of the significance of the number of reads of experiment versus control, indicating changes in expression levels. When a tile is selected, the gene is loaded underneath, scaled to fit the width of the screen. In a schematic view, all introns are shrunk to a fixed short length to visualise the aligned reads in a graph above the exons. To accomplish fast browsing, all reads are indexed on the server directly after data processing. This indexed data is also available for download to apply custom filtering in Excel or other programs. The export functions of the tiles and genes as scalable vector graphics makes it easy for the user to modify the final visualisation for publication.

### Workflow

**Fig. 1:**
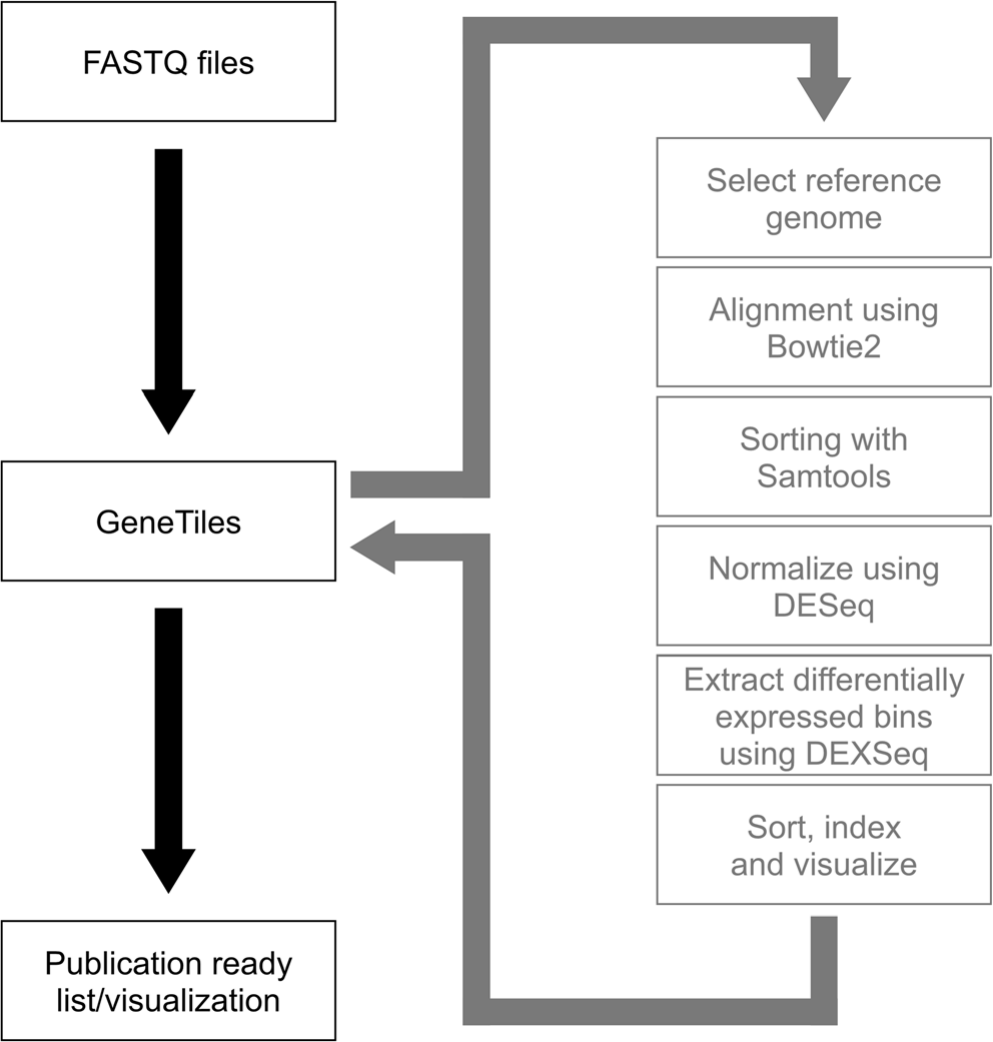
Pipeline of RNAseq data analysis. The diagram shows the workflow of the data analysis starting at the raw fastq files until the final visualisation performed automatically by the GeneTiles server. The analysis pipeline is an open source and available in the Supplement

Automated analysis of RNAseq data using GeneTiles does not need any programming steps anymore in a Linux environment by the user and performs directly a visualisation of the differentially expressed data, making it easier to interpret. To validate this new software package, RNAseq data from zebrafish bacterial infection experiments was obtained from Veneman et al. (2013) and used as the initial test model. All programs used by Veneman et al. (2013) are implemented, and more visualisation and export options are added in a server-based environment (Fig. 1, Online Resource 1). Therefore, the analysis pipeline of GeneTiles represents a combination of the previously described tools that have been previously shown to be useful for RNAseq analyses (Hatem et al. 2013). This makes it very manageable because it reduces the amount of high-end computers required in the research group for alignment and analysis, as all calculations are performed on the server. To start the analysis, the user can choose between various genomes that have been imported from Ensembl. Subsequently, the fastq files will be uploaded, followed by the option to analyse the data as single-end or paired-end. The files will be aligned automatically, after which the control or measurement treatment can be chosen. DESeq (Anders and Huber 2010) will normalise the data, and subsequently DEXSeq (Anders et al. 2012) will extract differentially expressed bins that indicate differential splicing. A table containing the differentially expressed genes or visualisations containing tiles or individual genes can be exported at this point.

With respect to the use of Bowtie2 aligner, we want to point out that it will fail to map reads spanning exon-exon boundaries to the genome. This problem could be solved using a splice aware aligner based on Bowtie2, such as Tophat2 (Kim et al. 2013). This option will be included in a future version of the GeneTiles package. However, Tophat2 is more computationally intensive and depends on the correct predictions of splice sites. Therefore, the analysis without the splice aware aligner, as used in this paper, will remain present.

### Different analysis methods

**Fig 2:**
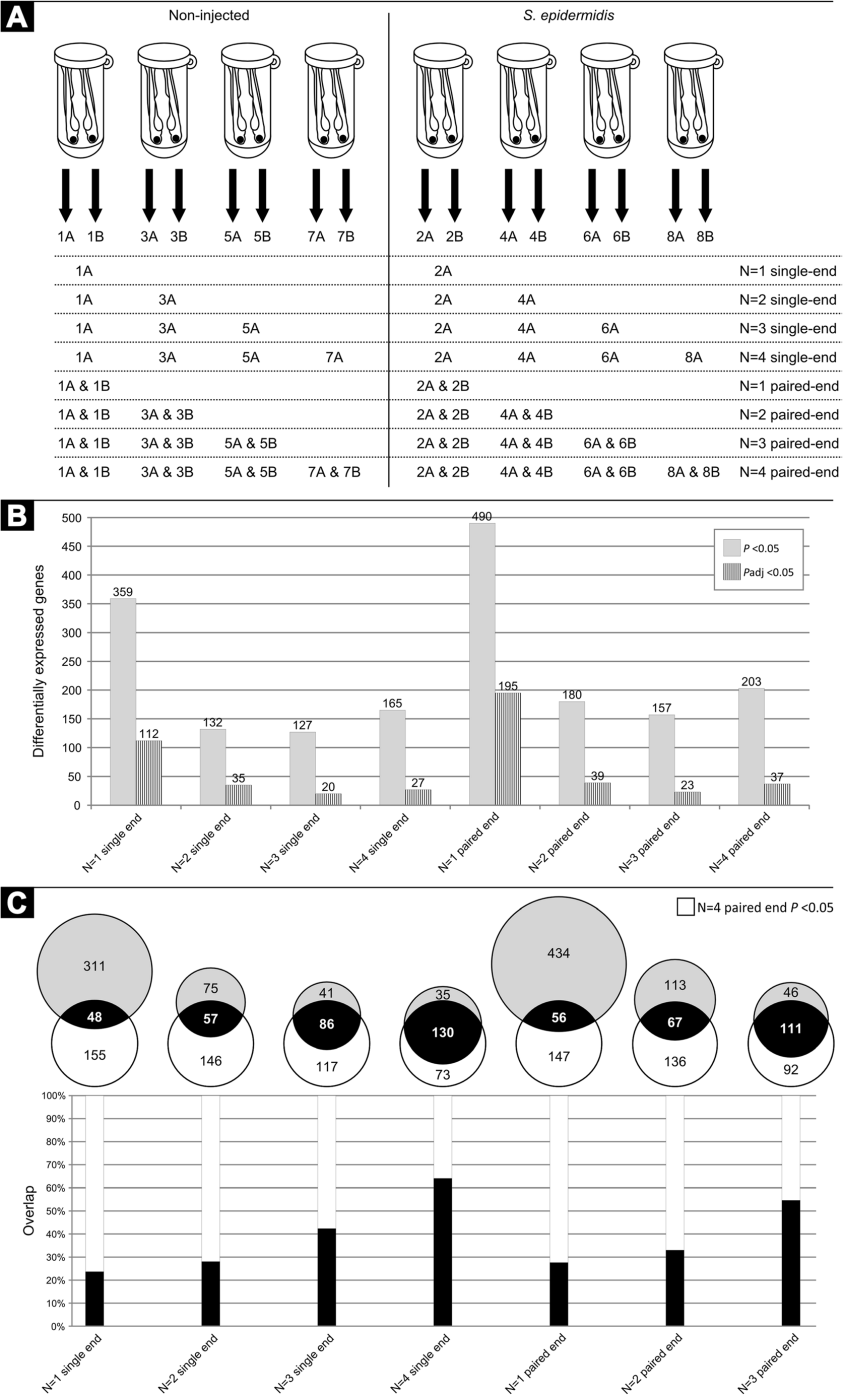
Comparing single- and paired-end RNAseq analysis. **a** The different sample sizes (*N* = 1–4) for analysis are visualised. **b** The total number of the differentially expressed genes with a fold change larger than 2 or smaller than −2 and a *P* value smaller than 0.05 (*solid grey bars*) or an adjusted *P* value smaller than 0.05 (*patterned dark bars*). **c** The *light grey circles* of the Venn diagrams show the total number of the differentially expressed genes for all the sample sizes with a fold change larger than 2 or smaller than −2 and a *P* value smaller than 0.05 as shown in (**b**), and the overlap of the differentially expressed genes compared to the *N* = 4 paired-end data set. The *bar graphs* show the overlap in percentage of these different sample sizes compared to the *N* = 4 paired-end data set

Considering that the RNA samples of the *S. epidermidis* infection experiments were paired-end sequenced, we had the possibility to explore the added value of paired-end over single-end sequencing. We compared the outcome of the differential expression of these two methods as well as the difference in sample sizes as shown in Fig. 2a. It can be noted that a the number of differentially expressed genes does not give an estimate of the reliability of the data; however, considering the high quality of RNAseq data, it can be assumed that adding an extra biological sample provides more relevant information than analysing a smaller group of samples by paired-end sequencing. Our data support this assumption since we found only a slight increase of 23 % in the differentially expressed genes when performing paired-end analysis in the four *S. epidermidis*-infected samples, using a *P* value of 0.05 as cut-off filter.

Secondly, we analysed the number of the differentially expressed genes with all possible options regarding samples sizes (Fig. 2b). We found a set of 359 differentially expressed genes when only analysing one sample with a *P* value of 0.05 and compared this large set of genes to our reference set of 203 genes (resulting from the analysis of paired-end sequence data of all four samples) (Fig. 2c). This comparison shows a rather small overlap of only 23.7 % (single-end) and 27.6 % (paired-end). As expected, this overlap increases, and therefore, the number of false-positives decreases when adding more samples (Fig. 2c). In order to provide a statistically more stringent analysis of the differentially expressed genes, we have also included, in the GeneTiles software, a tool for minimising false discovery based on the algorithm of Benjamini and Hochberg 1995 (Benjamini and Hochberg 1995), using the implementation of DESeq. The resulting adjusted values show far more stringent results (Fig. 2b) but generally confirm the limited value of performing paired-end sequencing as compared to the added value of adding more biological controls.

### Comparison of *S. epidermidis* versus *M. marinum* infection in zebrafish embryos

**Fig. 3:**
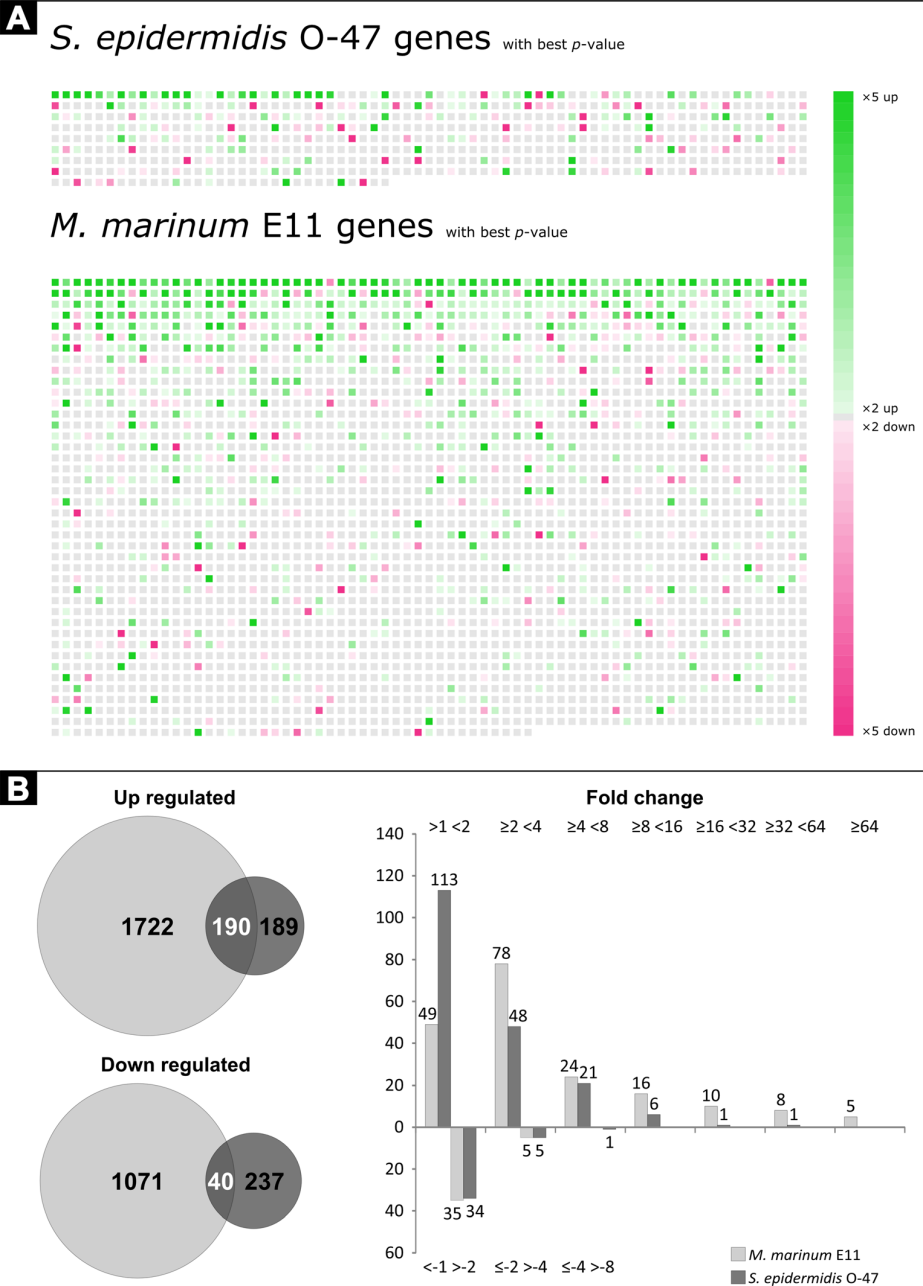
An overview of the differentially expressed genes. **a** The GeneTiles output shows a much larger set of genes with a *P* value smaller than 0.05 with the *M. marinum*-infected samples compared to the *S. epidermidis*-infected samples. Each tile visualises one gene, sorted on *P* value. The *colour and intensity* are a function of the ratio between measurement and control samples. **b** Comparing the data from 3A is shown in the Venn diagrams on the left and the overlaps in white digits are shown in a quantitative manner in the *bar graph* on the right

**Fig 4:**
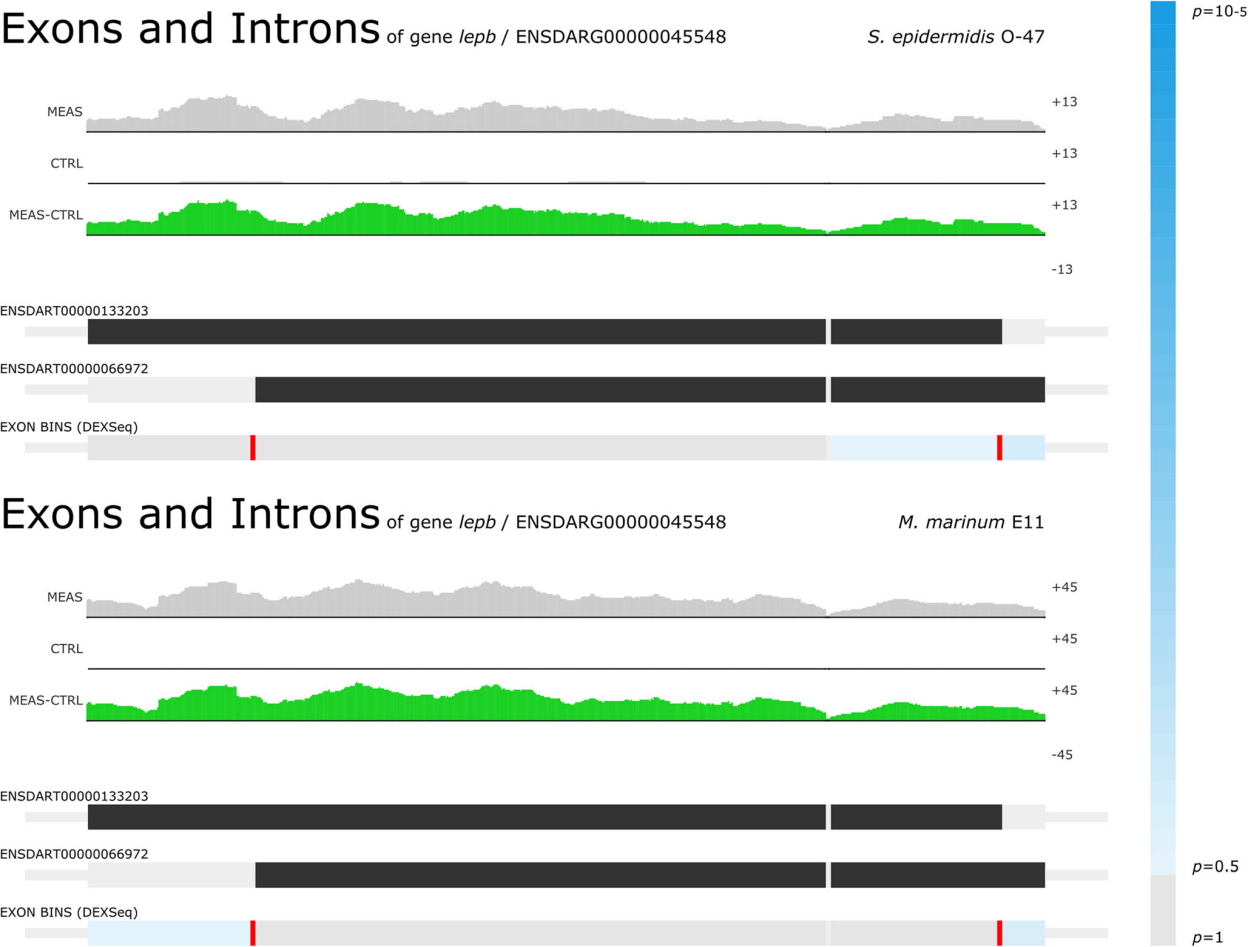
*Lepb* as the highest induced gene. For both S*. epidermidis* O-47-infected (FC 36, *P* value 4.99 × 10^−6^) and *M. marinum* E11-infected (FC 148, *P* value 1.54 × 10^−10^) samples, *Lepb* was found to be the highest induced gene

We compared the different transcriptome host responses of zebrafish embryos upon *S. epidermidis* or *M. marinum* yolk injection. The previous comparison as shown by Veneman et al. (2013) was based on a single biological replicate of *M. marinum*-infected zebrafish embryos. We used this single replicate and added 5 more independent biological replicas, which led to a total of 6 replicas of *M. marinum*, 4 replicas of *S. epidermidis*-infected zebrafish embryos and a total of 9 replicas of non-infected control samples. As found before (Veneman et al. 2013), *S. epidermidis* infection elicits a much smaller transcriptional host response of immune-related genes compared to *M. marinum* (Fig. 3). However, the total number of the differentially expressed genes was increased since several genes were previously not found to be significantly regulated by *S. epidermidis* where they do show a response in this analysis. Another finding is the high induction of genes upon *M. marinum* infection compared to the *S. epidermidis*-infected samples (Fig. 3b). An explanation could be that the *M. marinum* bacterium is a natural fish pathogen, and therefore, it is better recognised as intruder. *S. epidermidis* in large quantities is also pathogenic for fish; however, since it is not a natural pathogen, it could not be very well recognised as well as *M. marinum.* For instance, we now observe a high induction of the leptin B gene (*lepb*) upon infection with *S. epidermidis* (Fig. 4). In this case, the difference with the previous study is caused by errors in the automated annotation of probes in the micro-array used in Veneman et al. (2013), which was used as benchmark for the RNAseq analysis. The high expression of *lepb* found in *M. marinum*-infected samples is in line with the results earlier described by Wieland et al. 2005 (Wieland et al. 2005), where they found a higher mycobacterial load in the lungs of leptin-deficient *ob/ob* mice.

### Differential splicing

**Fig. 5:**
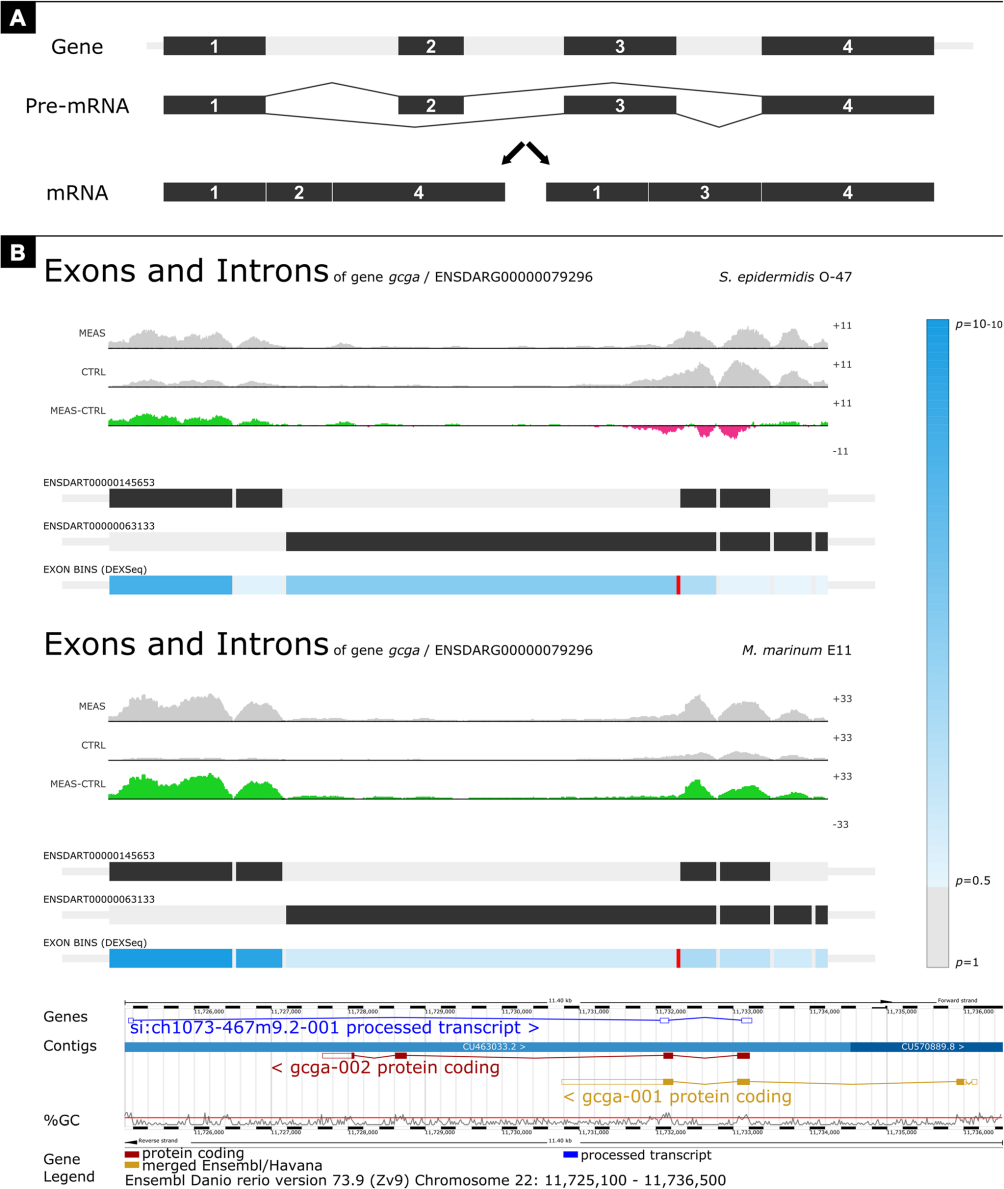
Finding the differentially spliced genes. **a** Schematic view of the principal differential splicing, where two different mRNAs are formed from one gene. **b** For both the *S. epidermidis-*infected (FC 1.17, *P* value 4.62 × 10^−1^) and *M. marinum*-infected (FC 4.70, *P* value 2.06 × 10^−10^) samples, glucagon A (*gcga*) was found to be differentially spliced as shown by the *dark blue bar* under the left exon. The screenshot of the GeneTiles visualisation demonstrates a schematic view, where introns are compressed to allow more space for visualisation of reads on exons. *The gradient-blue bar* on the right indicates the *P* value predicting the differential splicing. The bottom image is a gene representation from Ensembl

Another feature in the GeneTiles software is the integration of the DEXSeq analysis tool (Anders et al. 2012), which allows searching for genes that are differentially spliced. The analysis strategy of differential splicing is schematically shown in Fig. 5a, with an example that shows that 1 of 4 exons is spliced out from a pre-mRNA to form the mature mRNA. With both the *S. epidermidis* and *M. marinum* infection data sets, we found glucagon A (*gcga*) as top candidate to be differentially spliced with large enrichment of two 5′ exons (Fig. 5b), which are indicated by the *dark blue bars* underneath the representing exons. Supporting this finding, the pro-glucagon gene has been described before as being differentially spliced into multiple peptides in teleost fish (Holland and Short 2010).

**Fig. 6:**
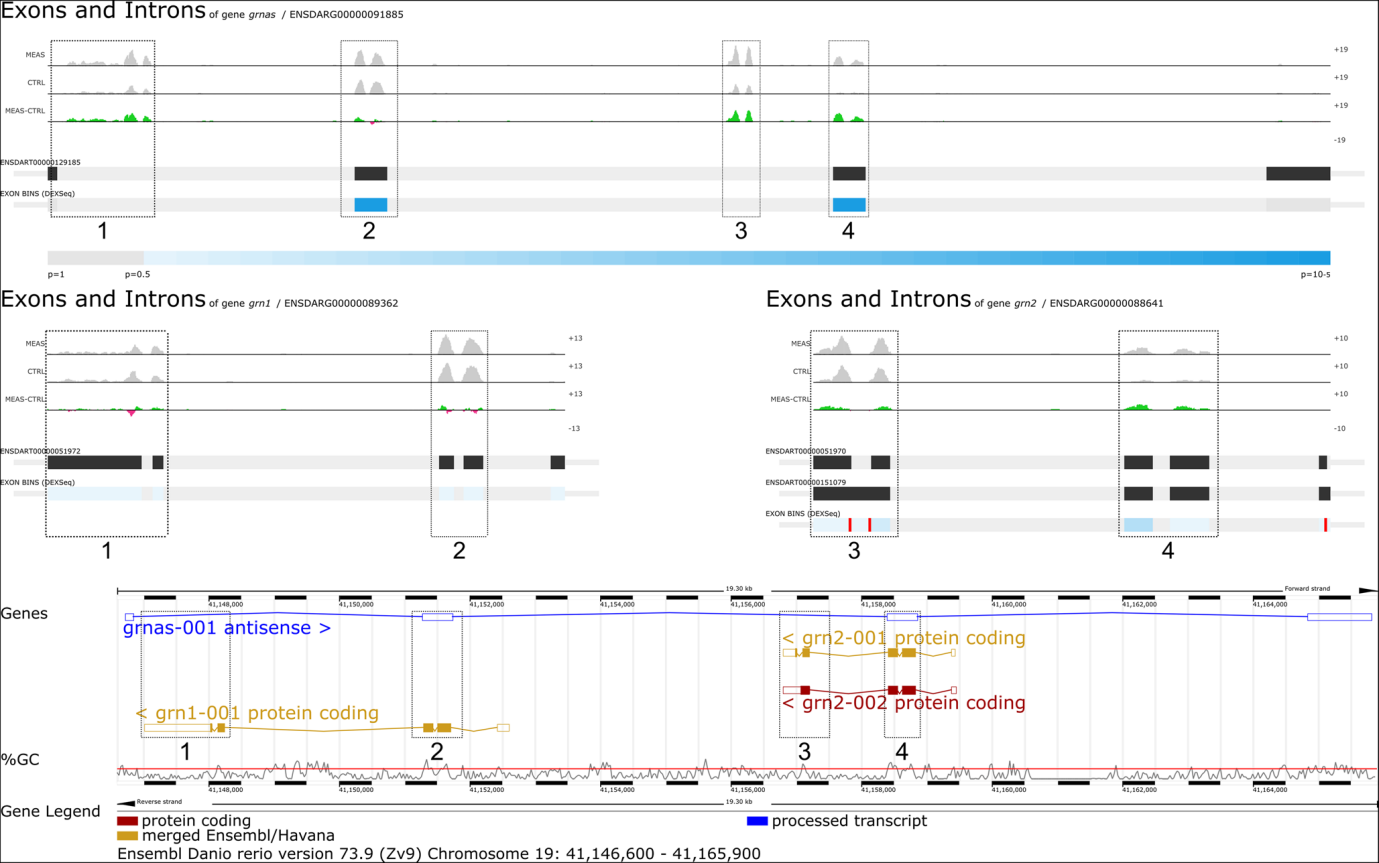
The differential splicing indicated by the *dark blue bars* at exon 2 and 4 from *grnas* proved to be incorrect. The differential expression found indicated by the 4 boxes at *grnas* (FC 1.87, *P* value 3.05 × 10^−3^) derived from *grn1* (FC 1.57, *P* value 1.80 × 10^−2^) and *grn2* (FC 3.33, *P* value 2.23 × 10^−3^). The screenshot of the GeneTiles visualisation demonstrates a non-schematic view, where introns are not compressed showing the actual length of the introns and exons. The *gradient-blue bar* indicates the *P* value predicting the differential splicing. The bottom image is a gene representation from Ensembl

We also demonstrate that GeneTiles can quickly point out false negative results based on DEXSeq as a result of ambiguous Ensembl annotations. For instance, in our analysis of infection markers, granulin antisense (*grnas*) appeared as a candidate for differential splicing. However, the actual differential expression found of *grnas* occurs from a fusion of two genes, granulin 1 (*grn1*) and granulin 2 (*grn2*), which are located at this same position as shown in Fig. 6.

### Comparing different host infection models

To date, we have only found 1 publication in PubMed describing RNAseq analysis of the host after a bacterial infection in vivo other than in zebrafish (Scholey et al. 2013). The data of Scholey et al. 2013 (Scholey et al. 2013), describing the bovine digital dermatitis (BDD, an infectious foot disease), was used to compare the host response in the cow and zebrafish. The raw data with the accession number GSE41732 was obtained from the GEO database and analysed with the GeneTiles software package. Comparing the differential expression data from Scholey et al. 2013 (Scholey et al. 2013), we could not validate all transcripts, since 23 % of the transcripts are retired and are not available anymore, due to the updated version of the *Bos taurus* 4.0 Ensembl annotation to the COW UMD3.1 Ensembl annotation. All other transcripts could be validated.

**Fig. 7:**
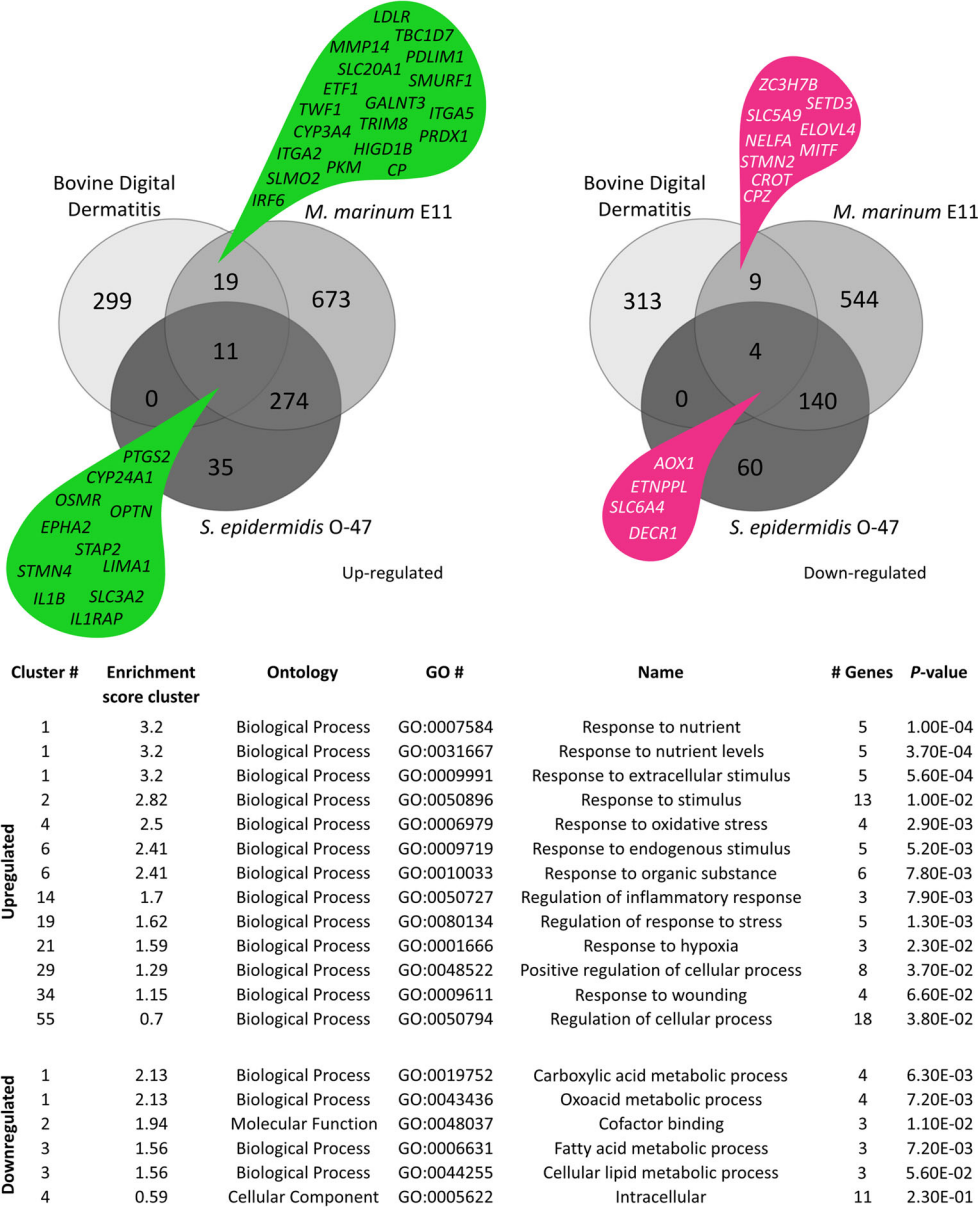
Overlap with other host pathogen RNAseq experiments. The Venn diagrams show the number of human orthologs of the differentially expressed (FC >2 or <−2, *P* value <0.05) genes from the bovine digital dermatitis, *M. marinum* E11 and *S. epidermidis* O-47 infection data. The gene ontology analysis (Huang da et al. 2009a; Huang da et al. 2009b) is based on the overlapping groups indicated by the *green and magenta drops*

Both zebrafish and bovine gene identifiers were linked to the human orthologs using the Ensembl database. The results show that 61 % of the zebrafish genes and 95 % of the bovine genes could be translated to human orthologs; a comparison of the differentially expressed gene sets in both disease models (*P* value 0.05) is shown in the Venn diagrams in Fig. 7. Gene ontology (GO) (Huang da et al. 2009a; Huang da et al. 2009b) analysis on the overlapping set of the differentially expressed genes between the cow and zebrafish showed that the differentially expressed genes are categorised in multiple response processes (Fig. 7). The group of upregulated overlapping genes between BDD, *M. marinum* and *S. epidermidis* infection includes the following genes: prostaglandin-endoperoxide synthase 2 (*PTGS2*); cytochrome P450, family 24, subfamily A, polypeptide 1 (*CYP24A1*); oncostatin M receptor (*OSMR*); optineurin (*OPTN*); EPH receptor A2 (*EPHA2*); signal transducing adaptor family member 2 (*STAP2*); stathmin-like 4 (*STMN4*); LIM domain and actin binding 1 (*LIMA1*); interleukin 1, beta (*IL1B*); solute carrier family 3 (amino acid transporter heavy chain), member 2 (*SLC3A2*) and interleukin 1 receptor accessory protein (*IL1RAP*). As expected, most of these genes are related to the immune system such as *OPTN*, which can activate Fas-ligand pathways to induce apoptosis or anti-inflammatory responses (Wild et al. 2011); and *IL1B* that is a well-known cytokine produced by activated macrophages, which then can indirectly activate *PTGS2* that also is significantly expressed (Lappas 2013). The *IL1RAP* is essential for signal transduction of *IL1* in order to induce proinflammatory proteins upon infection (Subramaniam et al. 2004).

### Pathway analysis

**Fig. 8:**
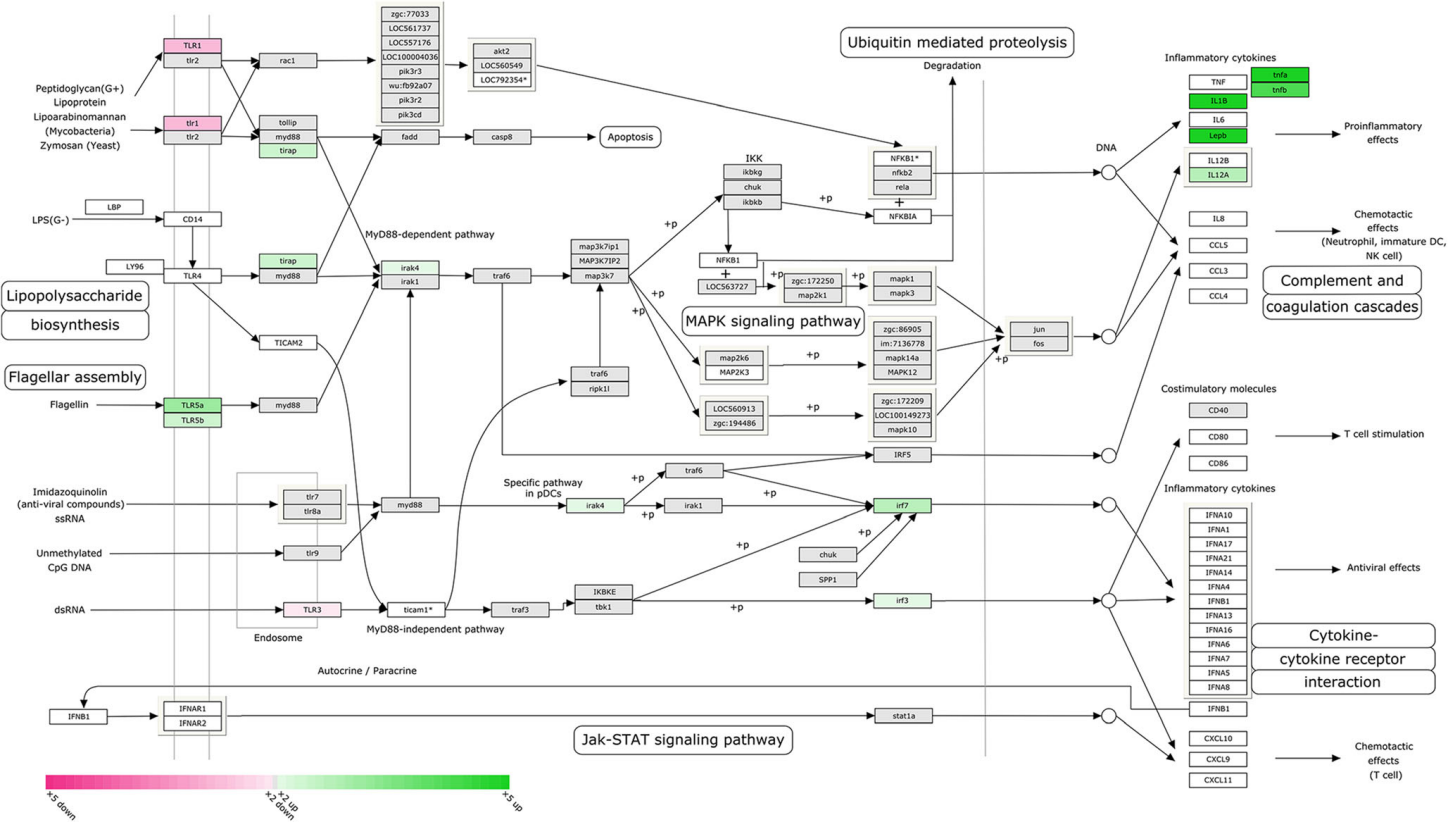
Toll-like receptor pathway showing *M. marinum* expression data. RNAseq expression data shows *M. marinum* E11-infected zebrafish at 5 dpi. The *green and magenta boxes* show up- and downregulation of the differential expression (FC >2 or <−2), respectively, with a *P* value smaller than 0.05. The asterisks show genes that are discontinued in current databases, the *white boxes* show genes that could not be identified and the *grey boxes* show genes that did not meet the expression criteria

The visualisation is not only limited to the coloured tiles as described above, but can also be used for functional analysis using WikiPathways (Kelder et al. 2012). With 96 zebrafish and 267 human pathways at the moment implemented in the software package, this allows the user a fast overview of differential expression in biological networks. An example is given in Fig. 8, where the toll-like receptor signalling pathway is showing the differential expression data of *M. marinum*-infected embryos using the pathway we submitted to WikiPathways that has been accepted in the curated collection.

## Conclusions

The described toolbox for RNAseq data analysis offers two different levels of support in an integrative setting. First, the software combines several programs needed for open source RNAseq analysis such as Bowtie2, Samtools, the ‘R’ statistical package, DESeq, DEXSeq, HTseq and pysam. These programs are placed in a pipeline (script) that runs these programs in the required order, with correct in- and output settings. Second, the processed data is visualised in a user-friendly way and made available for export with a choice of quantitative settings.

The advantages and ease of the use of this combined toolbox is demonstrated by analysis of the previously published RNAseq datasets from zebrafish and cow infectious disease models, as well as the new RNAseq data of a zebrafish mycobacterial infection experiments. This resulted in a highly confident innate marker set for systemic innate immune response to infection by pathogenic and non-pathogenic bacterial species in zebrafish. Furthermore, the data is viewable in a pathway view using the pathways stored at WikiPathways. In this way, it was also possible to quickly determine the effect of the number of replicates and the evaluation of potential false positive results, as is the case for the analysis of differential splicing using the DEXSeq algorithm. Comparing our experiences with our previous analyses (Veneman et al. 2013) and the re-analyses performed here, we can estimate that we have saved several months of working time while obtaining far superior output files that could be rapidly compared to the new RNAseq data sets also from other organisms. The data analysed in this study is available at the GeneTiles website for further analysis and as demonstration material. This makes it possible to rapidly evaluate new immune markers in the datasets described in this paper but also can be used to identify new markers based on other search criteria.

## Availability and requirements

The analysis is open source and available for download, as well as offered as Supplementary Material. All visualisation images are also available for download on the demo page of http://www.genetiles.com. The analysis pipeline, including the source and a complete script to run the same analysis locally, is available for download. It is offered together with the open demo for everyone, and it is possible to apply small changes locally to change the analysis or to select the input files.

## Electronic supplementary material

Below is the link to the electronic supplementary material.

ESM 1 Detailed representation of the GeneTiles server environment. From top to bottom: Add a new experiment, select the genome of interest, upload the raw RNAseq fastq files, align the files before starting the normalization, select the control or measurement treated samples and visualize or download the data. (PNG 3734 kb)

ESM 2 Detailed explanation of the GeneTiles pipeline. (DOCX 16 kb)

## References

[CR1] Anders S, Huber W (2010). Differential expression analysis for sequence count data. Genome Biol.

[CR2] Anders S, Reyes A, Huber W (2012). Detecting differential usage of exons from RNA-seq data. Genome Res.

[CR3] Anders S, Pyl PT, Huber W (2014). HTSeq—a Python framework to work with high-throughput sequencing data. Bioinformatics.

[CR4] Bao H, Guo H, Wang J, Zhou R, Lu X, Shi S (2009). MapView: visualization of short reads alignment on a desktop computer. Bioinformatics.

[CR5] Benjamini Y, Hochberg Y (1995). Controlling the false discovery rate—a practical and powerful approach to multiple testing. J R Stat Soc B Methodol.

[CR6] Boelens JJ (2000). Biomaterial-associated persistence of *Staphylococcus epidermidis* in pericatheter macrophages. J Infect Dis.

[CR7] Broekhuizen CA, Schultz MJ, van der Wal AC, Boszhard L, de Boer L, Vandenbroucke-Grauls CM, Zaat SA (2008). Tissue around catheters is a niche for bacteria associated with medical device infection. Crit Care Med.

[CR8] Busscher HJ (2012). Biomaterial-associated infection: locating the finish line in the race for the surface. Sci Transl Med.

[CR9] Carvalho R (2011). A high-throughput screen for tuberculosis progression. PLoS ONE.

[CR10] Carver T, Harris SR, Otto TD, Berriman M, Parkhill J, McQuillan JA (2013). BamView: visualizing and interpretation of next-generation sequencing read alignments. Brief Bioinform.

[CR11] Hatem A, Bozdag D, Toland AE, Catalyurek UV (2013). Benchmarking short sequence mapping tools. BMC Bioinforma.

[CR12] Holland LZ, Short S (2010). Alternative splicing in development and function of chordate endocrine systems: a focus on *Pax* genes. Integr Comp Biol.

[CR13] Huang da W, Sherman BT, Lempicki RA (2009). Bioinformatics enrichment tools: paths toward the comprehensive functional analysis of large gene lists. Nucleic Acids Res.

[CR14] Huang da W, Sherman BT, Lempicki RA (2009). Systematic and integrative analysis of large gene lists using DAVID bioinformatics resources. Nat Protoc.

[CR15] Kelder T, van Iersel MP, Hanspers K, Kutmon M, Conklin BR, Evelo CT, Pico AR (2012). WikiPathways: building research communities on biological pathways. Nucleic Acids Res.

[CR16] Kim D, Pertea G, Trapnell C, Pimentel H, Kelley R, Salzberg SL (2013). TopHat2: accurate alignment of transcriptomes in the presence of insertions, deletions and gene fusions. Genome Biol.

[CR17] Laczik M (2012). Geno viewer, a SAM/BAM viewer tool. Bioinformation.

[CR18] Langmead B, Salzberg SL (2012). Fast gapped-read alignment with Bowtie 2. Nat Methods.

[CR19] Lappas M (2013). NOD1 and NOD2 regulate proinflammatory and prolabor mediators in human fetal membranes and myometrium via nuclear factor-kappa B. Biol Reprod.

[CR20] Li H (2009). The Sequence Alignment/Map format and SAMtools. Bioinformatics.

[CR21] Milne I, Bayer M, Cardle L, Shaw P, Stephen G, Wright F, Marshall D (2010). Tablet—next generation sequence assembly visualization. Bioinformatics.

[CR22] Ordas A (2011). Deep sequencing of the innate immune transcriptomic response of zebrafish embryos to Salmonella infection. Fish Shellfish Immunol.

[CR23] Reyes A, Anders S, Huber W (2013) Inferring differential exon usage in RNA-Seq data with the DEXSeq package. http://www.bioconductor.org/packages/2.13/bioc/vignettes/DEXSeq/inst/doc/DEXSeq.pdf

[CR24] Robinson JT, Thorvaldsdottir H, Winckler W, Guttman M, Lander ES, Getz G, Mesirov JP (2011). Integrative genomics viewer. Nat Biotechnol.

[CR25] Scholey RA (2013). Identifying host pathogenic pathways in bovine digital dermatitis by RNA-Seq analysis. Vet J.

[CR26] Stockhammer OW, Rauwerda H, Wittink FR, Breit TM, Meijer AH, Spaink HP (2010). Transcriptome analysis of TRAF6 function in the innate immune response of zebrafish embryos. Mol Immunol.

[CR27] Subramaniam S, Stansberg C, Cunningham C (2004). The interleukin 1 receptor family. Dev Comp Immunol.

[CR28] van der Vaart M, van Soest JJ, Spaink HP, Meijer AH (2013). Functional analysis of a zebrafish myd88 mutant identifies key transcriptional components of the innate immune system. Dis Model Mech.

[CR29] van Soest JJ, Stockhammer OW, Ordas A, Bloemberg GV, Spaink HP, Meijer AH (2011). Comparison of static immersion and intravenous injection systems for exposure of zebrafish embryos to the natural pathogen *Edwardsiella tarda*. BMC Immunol.

[CR30] Veneman WJ, Stockhammer OW, de Boer L, Zaat SA, Meijer AH, Spaink HP (2013). A zebrafish high throughput screening system used for *Staphylococcus epidermidis* infection marker discovery. BMC Genomics.

[CR31] Wieland CW (2005). Pulmonary *Mycobacterium tuberculosis* infection in leptin-deficient *ob/ob* mice. Int Immunol.

[CR32] Wild P (2011). Phosphorylation of the autophagy receptor optineurin restricts Salmonella growth. Science.

[CR33] Zaat S, Broekhuizen C, Riool M (2010). Host tissue as a niche for biomaterial-associated infection. Future Microbiol.

