# Supplementary figures and images for: Analysis of RNAseq datasets from a comparative infectious disease zebrafish model using GeneTiles bioinformatics
Source: Immunogenetics. 2014 Dec 13;67(3):135–47. doi: 10.1007/s00251-014-0820-3 (PMC4325186; doi:10.1007/s00251-014-0820-3)

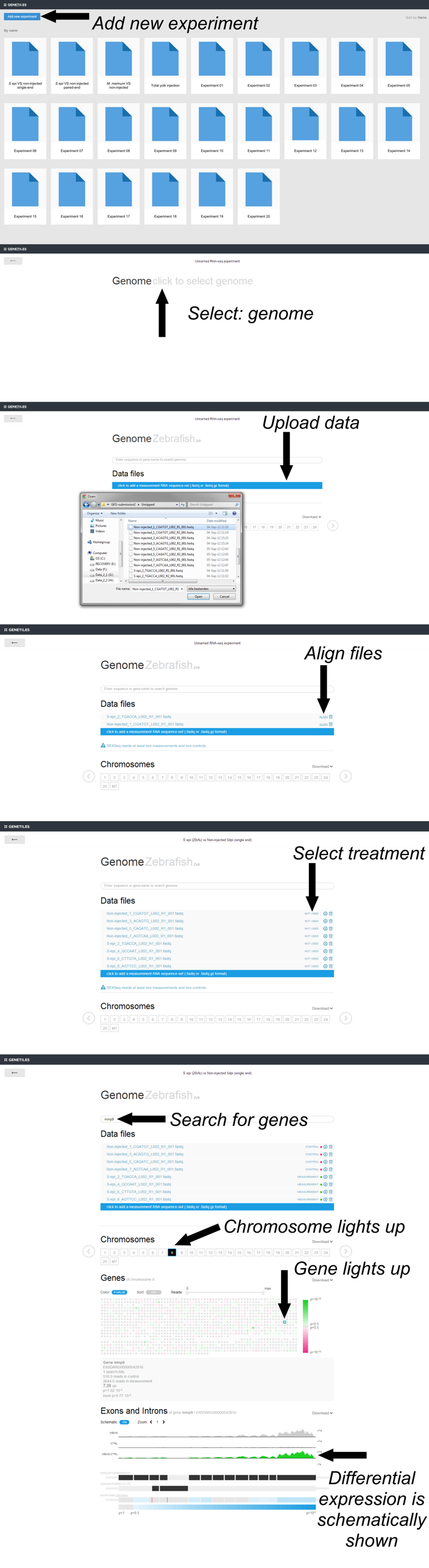

Supplement: Supplementary file 1 — Detailed representation of the GeneTiles server environment. From top to bottom: Add a new experiment, select the genome of interest, upload the raw RNAseq fastq files, align the files before starting the normalization, select the control or measurement treated samples and visualize or download the data. (PNG 3734 kb) [file 251_2014_820_MOESM1_ESM.png]
