# Supplementary material for: Analysis of RNAseq datasets from a comparative infectious disease zebrafish model using GeneTiles bioinformatics
Source: Immunogenetics. 2014 Dec 13;67(3):135–47. doi: 10.1007/s00251-014-0820-3 (PMC4325186; doi:10.1007/s00251-014-0820-3)
Supplement: Supplementary file 2 — Detailed explanation of the GeneTiles pipeline. (DOCX 16 kb) [file 251_2014_820_MOESM2_ESM.docx]

In order to run the commands in this section, the following software is required: the Linux operating system, Python, Perl, the “R” statistical package, bowtie2, samtools, wget. The following R packages are required: DESeq, DEXSeq. The following external Python packages are required: HTSeq, pysam.

We run bowtie2 for each input, as follows:

*bowtie2 -p 4 -q --phred33 -x genome -1 XXX.fastq -2 XXX.fastq –S 0.sam*

The resulting SAM files (containing the mapping information) are then converted to BAM format using the samtools program:

*samtools view -b -S -o N.bam N.sam*

Where N is the sample number. The BAM file is now sorted and indexed using these commands:

*samtools sort N.bam N.sorted*

*samtools index N.sorted.bam N.sorted.bam.bai*

In order to interpret these results, we need to have information about the location of genes, i.e., an annotation. We used annotation data from the Ensembl BioMart database, containing exon starting positions and lengths, and the relation between exons, transcripts and genes. We generated a table containing the read count per gene, using a Python script. This produced the file gene_counts.tsv, a file which follows the conventions of a spreadsheet with tab-separated format. See the DESeq manual for more details.

To perform DESeq, we run the following R script:

*datafile = "gene_counts.tsv"*

*count_table = read.table( datafile, header=TRUE, row.names=1 )*

*condition = factor( c("MEASUREMENT", "CONTROL", "MEASUREMENT", "CONTROL", "MEASUREMENT", "CONTROL", "MEASUREMENT", "CONTROL") )*

*library( "DESeq" )*

*cds = newCountDataSet( count_table, condition )*

*cds = estimateSizeFactors( cds )*

*write.table( sizeFactors(cds), file="size_factors.tsv", sep=" ")*

*cds = estimateDispersions( cds )*

*str( fitInfo(cds) )*

*res = nbinomTest( cds, "CONTROL", "MEASUREMENT" )*

*write.table( res, file="results.tsv", sep="\t")*

The results are available in results.tsv. The size-factors used for normalization are available in the file size_factors.tsv.

To generate *P*-values for differential splicing, we used the DEXSeq package, (Reyes et al. 2013). Using the DEXSeq instructions a python script was made to generate a gtf file. We generated the file containing the locations of the exons of all transcripts, and we converted that file to a flat format to make it suitable for DEXSeq. From here, we created the tables MN.txt. These tables contain the counts obtained for the mapping data in the .sam files, and the annotation in the .gff file. Finally, DEXSeq was invoked using R:

*library("DEXSeq");*

*design=read.table("dexseq_design.tsv", header=TRUE, row.names=1);*

*ecs = read.HTSeqCounts(countfiles=c("M0.txt", "M1.txt", "M2.txt", "M3.txt", "M4.txt", "M5.txt", "M6.txt", "M7.txt"), design=design, flattenedfile="genome.chr.gff");*

*ecs = estimateSizeFactors(ecs);*

*ecs = estimateDispersions(ecs);*

*ecs = fitDispersionFunction(ecs);*

*ecs = testForDEU(ecs);*

*ecs = estimatelog2FoldChanges(ecs);*

*res = DEUresultTable(ecs);*

*write.table(res, file="dexseq_results.tsv", sep="\t");*
